# Molecular Insights Into Memory-Enhancing Metabolites of Nicotine in Brain: A Systematic Review

**DOI:** 10.3389/fnins.2018.01002

**Published:** 2019-01-15

**Authors:** Alireza Majdi, Farzin Kamari, Saeed Sadigh-Eteghad, Albert Gjedde

**Affiliations:** ^1^Neurosciences Research Center, Tabriz University of Medical Sciences, Tabriz, Iran; ^2^Department of Clinical Research, University of Southern Denmark, Odense, Denmark; ^3^Department of Neuroscience, University of Copenhagen, Copenhagen, Denmark; ^4^Department of Neurology and Neurosurgery, McGill University, Montreal, QC, Canada; ^5^Department of Radiology and Radiological Science, Johns Hopkins University, Baltimore, MD, United States

**Keywords:** nicotine, metabolite, cotinine, norcotinine, nornicotine, cognition, systematic review

## Abstract

**Background:** The alleged procognitive effects of nicotine and its metabolites in brain are controversial.

**Objective:** Here, we review the pharmacologically active metabolites of nicotine in brain and their effects on neuronal mechanisms involving two main cognitive domains, i.e., learning and memory.

**Methods:** We searched Embase, Medline via PubMed, Scopus, and Web of Science databases for entries no later than May 2018, and restricted the search to articles about nicotine metabolites and cognitive behavior or cognitive mechanisms.

**Results:** The initial search yielded 425 articles, of which 17 were eligible for inclusion after application of exclusion criteria. Of these, 13 were experimental, two were clinical, and two were conference papers.

**Conclusions:** The results revealed three pharmacologically active biotransformations of nicotine in the brain, including cotinine, norcotinine, and nornicotine, among which cotinine and nornicotine both had a procognitive impact without adverse effects. The observed effect was significant only for cotinine.

## Introduction

The procognitive effects of nicotine are controversial. Some studies have shown positive effects of nicotine on learning and memory impairment in specific neurological disorders (López-Hidalgo et al., 2012; Newhouse et al., 2012; Allison and Shoaib, 2013; Majdi et al., 2018), while others reported negative effects of nicotine on cognitive abilities (Mundy and Iwamoto, 1988; Park et al., 2000).

After systemic administration, nicotine is extensively metabolized by the liver. Nicotine and some of its metabolites are biotransformed in the brain where they affect cognitive outcomes (Benowitz et al., 2009). Different metabolites of nicotine mediate different molecular and behavioral effects (Barreto et al., 2014), reported in studies of the influence of the metabolites on specific brain functions.

As the target of nicotine and its metabolites, nicotinic acetylcholine receptors (nAChR) modulate specific aspects of learning and memory (Majdi et al., 2017). Among different subtypes of nAChR, the α_7_ subtype may be mainly responsible for the procognitive and neuroprotective properties of acetylcholine (Sadigh-Eteghad et al., 2014; Wong et al., 2018). Although the metabolites of nicotine have lower affinity than nicotine to nAChR, the products interact with the receptors most likely as type 1 positive allosteric modulators (PAM) (Takeshima et al., 2007). The biotransformed metabolite cotinine long has been held to be responsible for memory supportive effects of nicotine, without the adverse effects (Green et al., 2000; Echeverria et al., 2011; Patel et al., 2014). In contrast, nornicotine has been found to possess the same addictive characteristics as nicotine (Green et al., 2000). Prevention of apoptosis, oxidative stress, and neuroinflammation, as well as augmentation of synaptic plasticity, modulation of glutamate release, and blockade of amyloid-beta or tau protein production pathways, are among the procognitive mechanisms proposed to underlie the effects of the metabolites (Soto-Otero et al., 2002; Hooper et al., 2008; Rehani et al., 2008; Echeverria et al., 2011; Moran, 2012), but the details of the molecular and behavioral mechanisms are incompletely understood.

Systematic reviews are tools that find relevant and unbiased answers to a research question (Sena et al., 2014). Due to the methodological strength, systematic reviews are reference standards for topics of controversy (Moher et al., 2015). The primary aim of this study was to identify known biotransformed products of nicotine in the brain, and the secondary aim was to reveal the known impacts on learning and memory and the mechanisms mediating the effects. First, we searched for specific biotransformed metabolites of nicotine in the brain, and second, we attempted to resolve the known effects on cognitive performance, including learning and memory and the mechanisms that mediate these brain functions.

## Methods

### Search Strategy

We electronically searched Embase, ISI Web of Science, MEDLINE via PubMed, and SCOPUS for studies that had investigated (1) nicotine metabolites in the brain as follows: [(nicotine)] AND [(metabolite)] AND [(brain) OR (central nervous system) OR (CNS)] and (2) the effects of nicotine metabolites on cognitive impairment as follows: [(memory) OR (learning) OR (cognition)] AND [(cotinine) OR (nicotine metabolite) OR (nornicotine) OR (nor-nicotine) OR (norcotinine) OR (nor-cotinine)]. Two investigators independently screened title, abstract and, where necessary, the full text, based on the inclusion and exclusion criteria. Where there were disagreements, the third investigator resolved the controversy. There was no date (all studies until May 2018) or species restriction in the search, but the search was limited to texts in English and original articles.

### Inclusion and Exclusion Criteria

We included all experimental and clinical studies reporting the effects of nicotine metabolites (i.e., cotinine, nornicotine, and norcotinine) as opposed to placebo or vehicle on learning and memory. Because cognition is a broad topic, and because evaluation of each domain requires comprehensive review, we focused on learning and memory in this systematic review, regardless of type or assessment task. All other domains of cognition were not investigated in this review. We excluded every study of the effects of smoking cigarettes, cigars, or pipe, or of ingesting tobacco in any form, on cognitive abilities. We also excluded studies that evaluated the effects of nicotine (rather than its metabolites) on the cognitive function. We examined the effects of nicotine in a previous publication (Majdi et al., 2017).

### Study Outcomes

The primary outcome of this review was evidence of specific biotransformed metabolites of nicotine in the brain, and the secondary outcome was evidence of effects on learning and memory and the mechanisms that mediate these brain functions.

### Data Extraction

From the included articles, we extracted data of the metabolites, the type of studies (clinical or experimental), the nature of the condition in which metabolites had effects, the actual effect(s) (positive or negative), and the mechanism, dose, duration, and route of metabolite administration. We also noted study quality measures to evaluate the risk of bias (see below).

### Quality of Selected Studies

A modified version of the CAMARADES' study quality checklist (Sadigh-Eteghad et al., 2017) was used to evaluate the methods used in the selected animal studies. The checklist provides the tools for assessment of the internal validity of the included studies (e.g., selection, performance, detection, and attrition bias) and other study quality measures (e.g., reporting quality and power). The items in the list include publication in a peer-reviewed journal, randomization to treatment or control, allocation concealment, blinded assessment of outcome, statement of inclusion and exclusion of animals from the study, sample-size calculation, statement of compliance with regulatory requirements and statement regarding possible conflicts of interest. The Cochrane risk of bias tool (Higgins et al., 2011) was used for human studies to determine different forms of bias, such as selection, performance, detection, attrition, and reporting.

## Results and Discussion

### Study Selection

**Figure 1:**
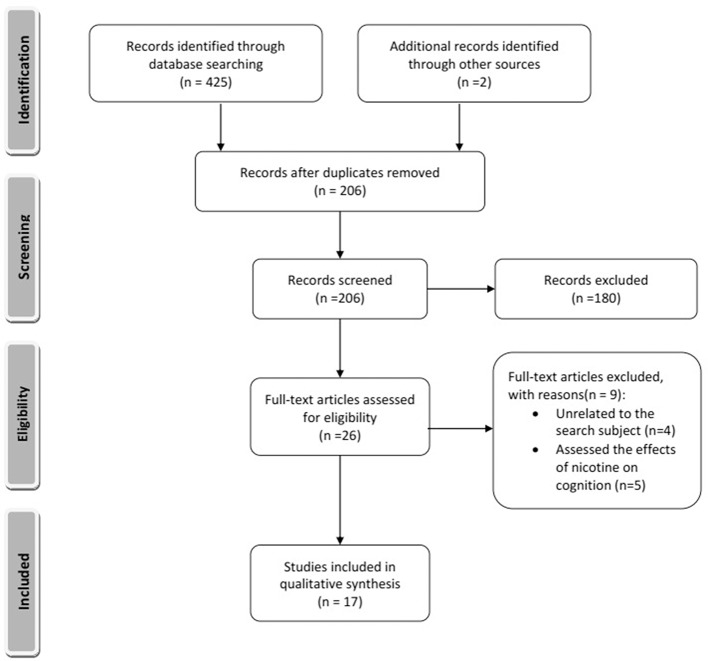
Summary of included and excluded articles. The style was adopted from Moher et al. (2009).

The electronic search of the mentioned databases identified 426 articles of which 17 studies met the inclusion criteria (Figure 1). Fifteen articles reported animal experiments, and two articles reported studies of humans. The search identified five nicotine metabolites in the brain including cotinine, nornicotine, norcotinine, and two unnamed minor metabolites that have not been characterized fully yet. All included articles addressed the effects of cotinine on learning and memory, and no study addressed the impact of other nicotine metabolites on cognition.

### Study Quality

**Figure 2:**
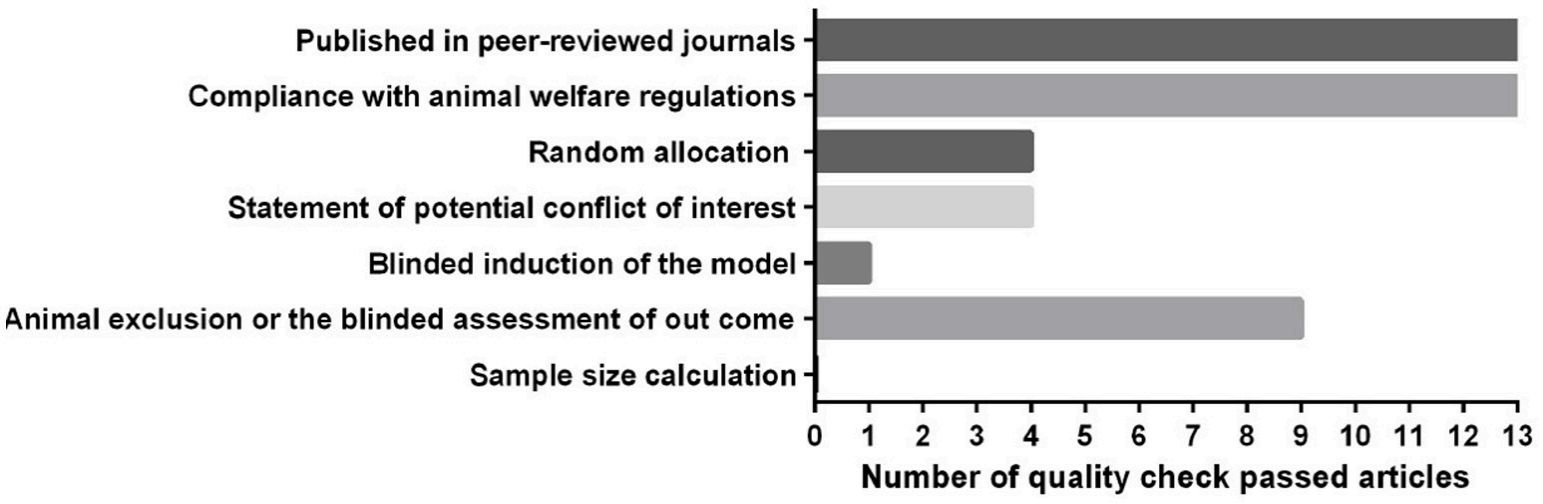
Quality assessment of the included animal studies according to modified CAMARADES' study quality checklist.

Low methodological quality of studies leads to overvaluation of effect sizes (Sadigh-Eteghad et al., 2017). We included 13 out of 17 publications into CAMARADES assessment. Two articles were human studies, assessed by the Cochrane tool, and two articles were conference papers that could not be evaluated by the checklists. The assessment showed that the quality of the animal studies included in the systematic review was modest (3.37 out of 8 items) (Figure 2). Some items on the checklist, such as reporting of animal exclusions, sample size calculation, and blinded induction of the model, usually were not reported. In contrast, the two human studies included in the review both had a low risk of bias. Considering the bias items in the design of future studies will reduce the risk of bias.

### Nicotine

#### Nicotine Metabolism

Upon delivery to the systemic circulation, nicotine is distributed throughout the body as ionized (69%) and unionized (31%) forms, and its binding to proteins is insignificant (Benowitz et al., 1982). The main organ of nicotine metabolism is liver, followed by kidney, spleen, and lungs. The metabolism of nicotine is also substantial in the brain, and due to the upregulation of nAChR, the metabolism in the brain is higher in smokers than in non-smokers (Hukkanen et al., 2005).

After distribution throughout the body, including the liver, nicotine is extensively metabolized by the liver, and the metabolites or the remaining nicotine are then excreted in the urine. A main first pass pathway of nicotine metabolism in the human liver is *C*-terminal oxidation to cotinine by cytochrome P450 2A6 (CYP2A6) which is the predominant metabolite (70–80%) of nicotine in mammals (Nakajima and Yokoi, 2005). Other metabolites of nicotine are nicotine *N*′-oxide (4–7%), nicotine glucuronide (3–5%), 4-oxo-4-(3-pyridyl) butanoic acid (1–2%), nicotine isomethonium ion (0.4–1%), and nornicotine (0.4–0.8%) (Byrd et al., 1992; Hukkanen et al., 2005). Cotinine is further metabolized by cytochrome P450 2A5 (CYP2A5), mainly to trans-3′-hydroxycotinine and its glucuronides (Ghosheh and Hawes, 2002; Kuehl and Murphy, 2003). Other metabolites of cotinine are 5′-hydroxycotinine, cotinine *N*-oxide, cotinine methonium ion, cotinine glucuronide, and norcotinine (Hukkanen et al., 2005). Nicotine, cotinine, and their metabolites are then excreted in the urine by kidneys (Meger et al., 2002).

#### Nicotine Metabolism in the Brain

Nicotine distributes to the brain shortly after peripheral administration (whether intraperitoneal, intravenous, oral, or subcutaneous) with maximum between 30 and 60 min, and can be detected in the CNS as late as 4 h after injection (Crooks and Dwoskin, 1997). In contrast to distribution after peripheral administration, smoking causes nicotine to massively distribute to the bloodstream and from there to the brain in 10–20 s (Majdi et al., 2017). Due to the prominence of the base and associated lipid solubility, nicotine readily penetrates the blood-brain barrier (BBB) at physiological pH (Oldendorf et al., 1979; Tega et al., 2013). In addition, nicotine is transported through the BBB as a monoprotonated cation by organic cationic transport systems (Tega et al., 2013). Upon administration of a single dose of nicotine (0.54 mg/kg) in rats, the following quantities of metabolites were found in the brain at 4 h post-injection: cotinine (44.6 ng/g brain), nornicotine (11.7 ng/g brain), and norcotinine (3.1 ng/g brain) (Crooks and Dwoskin, 1997).

Until recently, little attention has been paid to nicotine's metabolism in the central nervous system (CNS). The current urge to study nicotine and its metabolites in the brain arose from the evidence that the metabolites are pharmacologically active and may mediate nicotine's apparent effects in the brain (Crooks et al., 1995).

Besides nicotine, five metabolites of nicotine can be identified in the brain, including cotinine, norcotinine, nornicotine, and two minor *N*-demethylated metabolites that as yet have not been fully elucidated (Crooks et al., 1997; Ghosheh et al., 2001). They are either transported from the periphery, or they are the biotransformation products of nicotine in the brain (Crooks and Dwoskin, 1997; Ghosheh et al., 2001). The half-lives of the main metabolites (i.e., cotinine, norcotinine, nornicotine) significantly exceed their precursor's sojourn in the brain, and their concentrations are 6, 4, and 3 times higher than that of nicotine, respectively (Ghosheh et al., 2001). It has been shown that repeated peripheral administration of nicotine can cause significant accumulation of the metabolites in the brain that may contribute to the neuropharmacological effects of nicotine in the brain (Crooks et al., 1997; Dwoskin et al., 1999).

Although a large body of evidence supports the procognitive effects of nicotine, there is insufficient knowledge of the metabolites and their impact in the brain (White and Levin, 1999, 2004; Rezvani and Levin, 2001; Grizzell and Echeverria, 2015; Majdi et al., 2017). There is evidence that nicotine metabolites play a role in the positive neuropharmacological effects of nicotine (e.g., on memory and learning) in the brain (Crooks and Dwoskin, 1997), and the metabolites, and especially cotinine, do not show the common cardiovascular and addictive effects of nicotine in the tested subjects (Moran, 2012). Therefore, studies of the role of nicotine metabolites in the treatment of cognitive impairment have gained considerable attention.

### Cotinine

#### Cotinine Properties

Cotinine [(*S*)-1-methyl-5-(3-pyridinyl)-2-pyrrolidinone] is believed to be the main proximate metabolite of nicotine in the brain (Crooks et al., 1997). Structurally, it differs from nicotine only by an acetyl group (Fox et al., 2015). The accumulation of cotinine in the brain and its passage through the BBB are much slower than those of nicotine. Nicotine has been found to be present in the brain five min after subcutaneous injection, compared to cotinine's 30–60 min. The concentration peaks in 4 h and is detectable in the brain until 18 h after nicotine injection. As a result, its residence in blood and brain tissue is much longer than that of nicotine, and it may be responsible for nicotine's more prolonged pharmacological effects in the brain (Ghosheh et al., 1999; Buccafusco and Terry, 2003; Terry et al., 2005). Besides redistribution from the systemic circulation by passage through the BBB, some cotinine in the brain can also stem from local transformation of nicotine (Crooks and Dwoskin, 1997).

Cotinine does not cause tachyphylaxis, addiction, or nicotine-like withdrawal symptoms, and it has no negative cardiovascular effects as opposed to nicotine (Terry et al., 2005; Benowitz et al., 2009; Zeitlin et al., 2012). On the other hand, cotinine has positive effects on cognition and enhances learning, memory, and attention (Terry et al., 2005; Zeitlin et al., 2012). Therefore, as a pharmacologically active metabolite of nicotine, it may be a promising therapeutic option in the treatment of cognitive disorders (Terry et al., 2005).

#### Receptor Interactions

As a type 1 PAM of nAChR, cotinine's affinity is low compared to that of nicotine (Riah et al., 1999; Vainio and Tuominen, 2001; Takeshima et al., 2007). However, the affinity is high enough to trigger nicotinic responses in the brain (Vainio and Tuominen, 2001). Cotinine may enhance the effectiveness of endogenous ligands, such as acetylcholine but is unlikely to have agonist effects or to change the receptors' expression. In contrast to nicotine, cotinine does not interfere with receptor desensitization (Wildeboer-Andrud et al., 2014).

The findings cited above are not universally replicated, and some studies yielded opposite results. Rezvani and Levin (2001) showed that cotinine administration has the same effects as nicotine on the trafficking and assembly of nAChR and can up- or downregulate their expression, but at higher concentrations of cotinine, this effect appears to be lost. Although a majority of cotinine effects are mediated via α_7_ subtype, a recent study showed that chronic cotinine administration increases α_4_β_2_ subtype expression and the trafficking of receptors to the plasma membrane at doses around 1 μM, which equals its average blood concentration in a typical smoker. On the other hand, the highest doses (10 μM) was found to induce endocytosis and decrease α_4_β_2_ expression (Fox et al., 2015). More studies are needed to resolve the exact interactions between cotinine and nAChR fully.

#### Cognition

**Figure 3:**
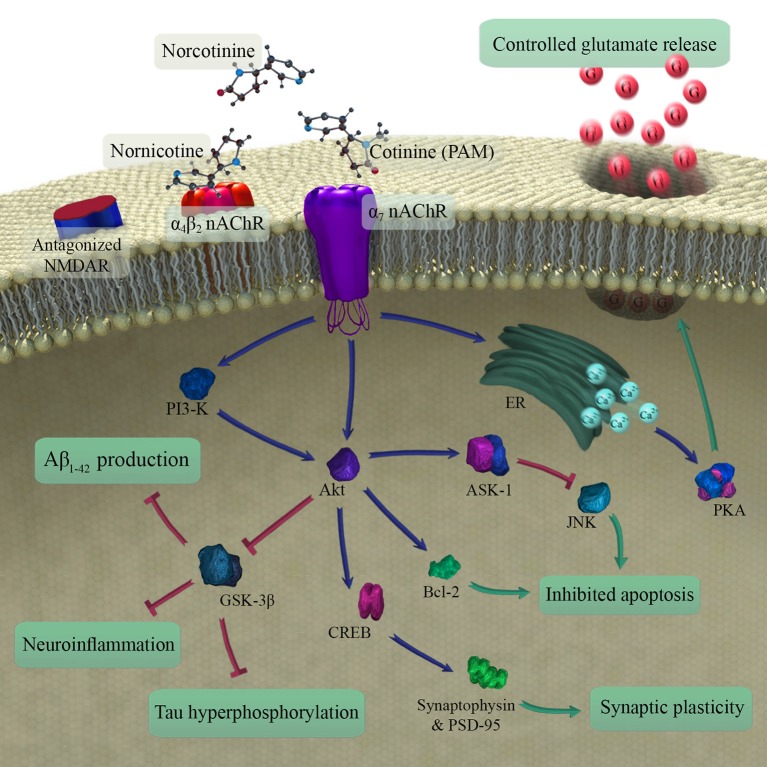
Schematic illustration of metabolites of nicotine in brain and mechanisms involved in the procognitive effects of cotinine (the main procognitive metabolite). As a type 1 PAM, cotinine modulates the function of α_7_ nAChR that in turn leads to reduced Aβ_1−42_ production and decreased neuroinflammation, tau hyperphosphorylation, and apoptosis. It also improves synaptic plasticity. In the end, the changes may contribute to the reduction of age-related cognitive impairment. PAM, positive allosteric modulator; nAChR, nicotinic acetylcholine receptor; NMDAR, N-methyl-D-aspartic acid receptor; ER, endoplasmic reticulum; PKA, protein kinase A; Aβ, amyloid-beta.

A growing body of evidence supports a procognitive effect of cotinine in animals (Herzig et al., 1998; Grizzell et al., 2014a; Grizzell and Echeverria, 2015). However, the two studies of cognition in humans included here failed to replicate the positive effects of cotinine on the cognitive performance of animals (Hatsukami et al., 1997; Herzig et al., 1998) (Table 1). The discrepancy may stem from the fact that neither human study examined the effects of chronic cotinine administration on human subjects, with cotinine administered for either 1 or 3 days. Thus, chronic cotinine administration in clinical studies deserves further investigation. Also, interspecies differences between rodents and humans may justify the observed differences among studies. The limited qualities of experimental studies and the lack of vigorous designs may also play a role in this regard. Figure 3 illustrates the major pathways found to mediate procognitive effects of cotinine. The material discussed in the following sections is based on evidence from animal studies.

**Table 1:** Selected studies investigating the effects of cotinine on cognitive performance in various neurological disorders.

**Species**	**Type of disease**	**Effect(s)**	**Mechanism(s)**	**Dose**	**Duration**	**Route**	**References**
Mouse	Tg6799 Model of AD	Prevents memory loss	Reduction of Aβ aggregation and stimulation of the Akt/GSK3β pathway	2.5 mg/kg	3.5 months	Oral gavage	Echeverria et al., 2011
		Improved spatial working memory	Lowering Aβ burden in the hippocampus and entorhinal cortex	5 mg/kg	3 months	Oral gavage	Patel et al., 2014
		Improved visual recognition memory	Changes in the cerebral Tau phosphorylation	5 mg/kg	3.5 months	Oral gavage	Grizzell et al., 2017
	Model of chronic stress	Enhanced learning and memory	Improvement of the expression of the neurogenesis factor VEGF	5 mg/kg	13 days	Oral gavage	Grizzell et al., 2014b
		Enhanced working memory impairment	Increase in the synaptic density and activates the Akt/GSK3β pathway in hippocampus	5 mg/kg	37 days	Oral gavage	Grizzell et al., 2014a
		Improved memory	Enhancement of expression of GFAP in the hippocampus and frontal cortex of mice	10 mg/ml	2 weeks	Intranasal	Perez-Urrutia et al., 2017
	PTSD model	Improved the extinction of fear memory	Increase in the levels of the active forms of ERK1/2	5 mg/kg	NM	Oral gavage	Zeitlin et al., 2012
		Prevented working memory loss induced by model of chronic stress	Increase in the synaptophysin, in the CA1 region of hippocampus, entorhinal and prefrontal cortices	5 mg/kg	3 weeks	Oral gavage	Alex Grizzell et al., 2012
	Model of Fragile X syndrome	Improved coordinate and categorical spatial processing, novel object recognition, and temporal ordering	Increase in the phosphorylation of GSK3β and Akt in the hippocampus	3 mg/kg	Acute	Intraperitoneal	Pardo et al., 2017
	DBA/2 model of sensory inhibition deficit	No improvement of sensory inhibition	Probable activation of α_7_ nAChR on hippocampal interneurons and also α_4_β_2_ activation	0.033, 0.1, 0.33, 1, 3.3 mg/kg	Single dose	Subcutaneous	Wildeboer-Andrud et al., 2014
				0.33, 1, 3.3 mg/kg	7 days		
Rat	NMDAR-blocked dementia model	Improved recognition memory	Attenuation of NMDA antagonist-induced memory impairment	2 mg/kg	Chronic	Oral gavage	Terry et al., 2011
		Improved working memory	Attenuation of NMDA antagonist-induced memory impairment	0.03–10.0 mg/kg 2.0 mg/kg	Single dose	Subcutaneous	Terry et al., 2012
					Chronic	Oral gavage	
	Healthy	Improved the extinction of fear memory	Increase in pERK/tERK ratios and pERK 1/2 (without impairment of cognition)	2.0 mg/kg	Chronic	Oral gavage	de Aguiar et al., 2013
	Healthy	Enhanced recognition memory	Sensitize α_7_ nAChR to low levels of acetylcholine	3.0 and 10.0 mg/kg	Single dose	Intraperitoneal	Terry et al., 2015
	Chemotherapy model	Improved working memory	Probable modulation of α_7_ nAChR	5 mg/kg	2 weeks	Oral gavage	Iarkov et al., 2016
Human	Non-smokers	Impaired verbal recall on the long word list	no data	0.5, 1, and 1.5 mg/kg	Single dose	Oral capsule	Herzig et al., 1998
	Abstinent cigarette smokers	No significant effects in symbol digit modalities test	no data	40, 80, or 160 mg/daily	10 days	Oral capsule	Hatsukami et al., 1997

*AD, Alzheimer's disease; VEGF, vasoendothelial growth factor; Aβ, amyloid-beta; GSK3β, glycogen synthase kinase 3 beta; NMDAR, N-methyl-D-aspartate receptor; PTSD, post-traumatic stress disorder; nAChR, nicotinic acetylcholine receptor*.

##### Apoptosis and neuronal survival

Apoptosis, a programmed form of cell death, has been implicated in the pathogenesis of memory disorders, such as AD (Majdi et al., 2016). This process is controlled by a variety of pro- and anti-apoptotic signals inside neurons (Kim et al., 2001). Akt is a family of serine-threonine-specific protein kinases that inhibit programmed cell death and promote neuronal survival by phosphorylation and inhibition of proapoptotic proteins, such as glycogen synthase kinase 3 (GSK3) (Dudek et al., 1997; Kim et al., 2001). Cotinine, by its positive allosteric effects on α_7_ nAChR, activates the Akt pathway that subsequently raises the expression of anti-apoptotic proteins, such as the cAMP response element binding (CREB) protein and B-cell lymphoma protein 2 (Bcl-2). Akt stimulation also decreases the activity of pro-apoptotic factors including c-Jun N-terminal kinase (JNK) by triggering apoptosis signal-regulating kinase 1 (Ask-1) that ultimately promotes neuronal survival (Kim et al., 2001; Moran, 2012).

##### Synaptic plasticity and density

Synaptic plasticity and density are of central importance to learning and memory (Silva, 2003). Studies prove that synaptic dysfunction happens before neuronal degeneration in neurodegenerative disorders and age-related cognitive decline (Selkoe, 2002; VanGuilder et al., 2011; Phan et al., 2017). A marker of synaptic density, synaptophysin is detected in synaptic vesicles (Valtorta et al., 2004). Cotinine has been shown to remarkably increase the expression of synaptophysin, and with it, synaptic density in the prefrontal cortex and hippocampus and thus to improve learning and memory (Grizzell et al., 2014a). An increase in the expression of post-synaptic density protein-95 (PSD-95), which also promotes synaptic plasticity, has been reported with cotinine treatment. The mechanism of both of these changes is the cotinine-induced modulation of α_7_ nAChR that subsequently stimulates protein kinases phosphoinositide-3 kinase (PI3K). PI3K then induces Akt phosphorylation, leading to increase in the CREB protein transcriptional activity. The increase raises the expression of the synaptic proteins and improves cognitive performance (Zeitlin et al., 2012; Grizzell et al., 2014b).

##### Amyloid-beta production and aggregation

As the main neurotoxic forms of Aβ, amyloid-beta_1−42_ (Aβ_1−42_) oligomers are believed by some to cause the cognitive dysfunction of AD (Resende et al., 2008; Sadigh-Eteghad et al., 2015). Cotinine blocks Aβ_1−42_ aggregation and oligomerisation, reduces number and size of plaques, decreases the Aβ_42_/Aβ_40_ ratio. Protection of neurons against Aβ_1−42_-induced neurotoxicity and possible subsequent improvement of cognition (Burgess et al., 2011; Echeverria et al., 2011) are not explained by interaction with nAChR, as the effects are not eliminated by blockade of the receptors (Burgess et al., 2011). The mechanism of cotinine's effects on the Aβ clearance, therefore, remains unclear, although cotinine inhibits activation of GSK3β and may reduce Aβ_1−42_ production by Akt activation in both cortex and hippocampus (Echeverria et al., 2011). GSK3β is a proline-directed serine-threonine kinase, and excessive activation may impair memory by increase of Aβ production and hyperphosphorylation of tau (Hooper et al., 2008).

##### Tau hyperphosphorylation and NFT formation

Hyperphosphorylated tau is the major component of neurofibrillary tangles (NFT) that are a key pathological finding in AD and other cognitive disorders (Mitchell et al., 2002). Tau accumulation in the temporal lobe correlates better with cognitive dysfunction than Aβ deposition in any region of the brain (Brier et al., 2016). Tau is phosphorylated by GSK3β, and this enzyme's activity is closely associated with NFT burden in AD brains (Baum et al., 1996; Plattner et al., 2006). As discussed above, cotinine inhibits tau hyperphosphorylation through activation of the Akt pathway and subsequent blockade of GSK3β in a concentration-dependent manner. Evidence suggests that α_7_ nAChR may mediate these effects of cotinine on the brain (Burgess et al., 2008; Echeverria et al., 2011).

##### Modulation of glutamate release

Controlled release of glutamate in the cortex regulates high cortical functions, such as learning and memory (Rahn et al., 2012), and disruption of glutamatergic neurotransmission has been implicated in the pathogenesis of cognitive decline (Tsai and Coyle, 2002). It has been shown that cotinine administration enhances attention and executive function in glutamate antagonist-induced cognitive impairment in the rat, possibly due to the activation of α_7_ nAChR (Terry et al., 2012).

Activation of α_7_ receptors stimulates calcium release from intracellular sources (Dajas-Bailador et al., 2002). Also, α_7_ nAChR enhance depolarization of nerve terminals, opening voltage-gated calcium channels with calcium entry into the cell. Increased calcium levels then, directly and indirectly, raise glutamate release from synapses through activation of cAMP-PKA-dependent pathways (Girod et al., 2000; Cheng and Yakel, 2015). The α7 nAChR induced glutamate surge also plays a role in presynaptic facilitation and synaptic plasticity (Livingstone et al., 2010).

##### Neuroinflammation

The anti-inflammatory properties of nAChR, especially the α_7_ subtype, are well-known from numerous studies (Metz and Tracey, 2005; de Jonge and Ulloa, 2007; Egea et al., 2015). Neuroinflammation is a hallmark both of normal brain aging and of pathological aging with cognitive disorders, such as AD (Ownby, 2010; Sadigh-Eteghad et al., 2016), and elevation of inflammatory markers is directly linked to the degree of cognitive impairment (Ownby, 2010). Through an nAChR and NF-κB-dependent pathway, cotinine lowers the levels of pro-inflammatory molecules, such as TNF-α, IL-1β as well as IL-6 and enforces anti-inflammatory cytokines including IL-10 production (Rehani et al., 2008). Also, cotinine exerts its anti-inflammatory effects via regulation of PI3K-Akt and inhibition of the GSK3β pathways that provoke neuroinflammation (Rehani et al., 2008; Echeverria et al., 2016). This action makes cotinine a potential candidate for the treatment of the neuroinflammatory disorders, e.g., as seen in AD.

##### Oxidative Stress

Under controlled circumstances, cotinine blocks Fenton's reaction and prevents free radical production in the brain (Soto-Otero et al., 2002). Evidence suggests that addition of cotinine and iron to the media before H_2_O_2_ blocks free radical formation and reduces oxidative stress. This can be partly explained by the fact that addition of nicotine or cotinine chelates iron and halts Fenton's reaction (Nakajima et al., 1996; Soto-Otero et al., 2002). It appears that cotinine also lowers lipid peroxidation in a manner that cannot be explained by the effects on Fenton's reaction. The reduction can result from chain-breaking antioxidant properties of cotinine (Soto-Otero et al., 2002). Oxidative stress and lipid peroxidation are of crucial importance to brain aging and neurodegeneration and the accompanying cognitive decline (Sadigh-Eteghad et al., 2015; Pourmemar et al., 2017). Thus, treatment with anti-oxidant effect is a top priority in these conditions (Fukui et al., 2002; Mecocci, 2004; Williams et al., 2006; Schrag et al., 2013).

### Nornicotine

Nornicotine or demethylcotinine is a major pharmacologically active metabolite of nicotine in the brain which possibly acts via nAChR (Dwoskin et al., 2001). Oxidative *N*-demethylation of nicotine is the major pathway by which nornicotine is produced in the CNS (Crooks et al., 1997; Ghosheh et al., 2001). Compared with the periphery where nornicotine is considered to be a minor metabolite (0.8%), its concentration in the brain is higher for several reasons, including the longer half-life in comparison to nicotine, its superior partitioning as well as active transport to the CNS and transformation of nicotine to nornicotine in the brain (Ghosheh et al., 2001).

Although nornicotine is as potent as nicotine, it is less desensitizing at the major nAChR subtypes in the brain, and nornicotine's presence leads to the activation of α_7_ nAChR. Nornicotine's potency and efficacy differ by several folds, but it has been shown that peak currents caused by nornicotine acting at α_7_ nAChR are equal to those of acetylcholine. Considering nornicotine's durable presence in the brain, the molecule may mediate some of the neuroprotective effects of nicotine. A study showed that α_7_ receptors are responsive to nornicotine, and the action at the receptors of this nicotine metabolite leads to improved cognition and attention (Papke, 2006; Papke et al., 2007). Nornicotine may also alter Aβ's aggregation, possibly via reduced plaque formation or altered clearance of the peptide, or both, as well as by attenuated toxicity of soluble Aβ aggregates (Dickerson and Janda, 2003). More studies are needed to better define nornicotine effects on brain function, learning, and memory.

### Norcotinine

In addition to the major metabolites mentioned above, there are minor CNS biotransformation products of nicotine, including norcotinine. After peripheral injection of nicotine, norcotinine is detected in the brain, and it is likely produced by 5′-C-oxidation of brain nornicotine. This fate is different from the processing in the periphery where *N*-demethylation of cotinine produces norcotinine. It has been shown that only 0.16% of cotinine is metabolized into norcotinine (Li et al., 2015). *In vivo*, the metabolite neither evoked the release of dopamine from rat striatal slices nor inhibited dopamine uptake into rat striatal synaptosomes (Crooks et al., 1995), suggesting that this minor metabolite, in fact, may be pharmacologically inactive (Crooks et al., 1997). Thus, there is no information on norcotinine's effects on cognitive performance, but possible effects are under investigation because of the pharmacological and therapeutic potentials of cotinine in cognitive disorders, such as AD (Li et al., 2012).

## Conclusion

Nicotine lowers learning and memory impairment in some neurological disorders. However, its adverse cardiovascular and addictive effects limit the application in the clinical setting. Possible biological effects of nicotine in the human brain in principle could be mediated by nicotine itself or by its metabolites, but there is a considerable lack of evidence of the mechanistic effects of specific compounds in humans. This shortage of evidence can be rectified only by focused research in the future. On the other hand, evidence suggests that the biotransformation product cotinine is pharmacologically active in the brain of animal models with no adverse effects. Accumulating evidence makes it likely that this metabolite mediates the memory supportive effects of nicotine in the brain. Thus, a great deal of effort has been exerted to clinically apply cotinine as a treatment of learning and memory impairment and its underlying disorders. Taken together, we claim that this biologically active metabolite is more than just a biomarker of nicotine consumption and has potentially novel therapeutic value in the treatment of learning and memory declines.

## Author Contributions

AM, FK, and SS-E performed the searches, interpreted the results and wrote the manuscript. AG, SS-E, and AM designed the study. AG critically interpreted data and critically revised and approved the manuscript.

### Conflict of Interest Statement

The authors declare that the research was conducted in the absence of any commercial or financial relationships that could be construed as a potential conflict of interest.
